# Characterization of bacterial community in tobacco leaves at flue-curing and redrying processing stages

**DOI:** 10.1038/s41598-023-40502-0

**Published:** 2023-08-16

**Authors:** Yue Yang, Ruyan Xu, Mengmeng Yang, Qiang Xu, Chenlin Miao, Jianhua Guo, Wenjun Mou, Hang Du, Gang Wei, Liwei Hu, Zongyu Hu

**Affiliations:** 1 China Tobacco Jiangsu Industrial Co., Ltd., Nanjing, 210000 Jiangsu China; 2 Zhengzhou Tobacco Research Institute of CNTC, Zhengzhou, 450001 Henan China

**Keywords:** Microbiology, Molecular biology

## Abstract

During the processing of tobacco leaves, flue-curing and redrying can affect the structure of bacterial community, having an effect on the aging quality of tobacco leaves. In order to characterize the effects of flue-curing and redrying on the bacterial community of tobacco leaves, the bacterial community of samples at different processing stages (before flue-curing, after flue-curing, before redrying and after redrying) was analyzed using Illumina sequencing. A total of 33 phyla, 79 classes, 195 orders, 344 families, 826 genera and 7922 ASVs were obtained from 36 samples. There was no significant difference in the core bacterial groups of tobacco leaf at four processing stages. *Proteobacteria* dominated at the phylum level. *Sphingomonas*, *Pseudomonas* and *Methylobacterium* were the main genera shared by all samples. The functional prediction by PICRUSt showed an increase in the relative abundance of pathway related to metabolism after flue-curing and pathway related to environmental information processing after redrying. This study, we analyzed the changes of bacterial community and structural composition of tobacco leaves from flue-curing to redrying, and found that flue-curing had a greater effect on the microbial community than redrying. This is conducive for the exploration of microbial resources and improvement of tobacco leaf quality.

## Introduction

Tobacco is an important economic crop in China and also many other countries in the world. Freshly picked tobacco leaves have defects in quality such as impurities, high irritation and undesirable flavor^1,2^. In order to improve the quality, harvested tobacco leaves needs to undergo processing stages such as flue-curing, redrying, and aging^3,4^. High temperature treatment and severe physical shearing during the flue-curing and redrying process may affect the microbial community structure on the surface of tobacco leaves, which play an important role in the aging process of tobacco leaves^5^. Therefore, flue-curing and redrying are key steps in determining the quality and yield of tobacco leaves. It is important to investigate the composition and dynamics of bacterial community during tobacco leaf processing^6,7^.

High throughput sequencing technology, known as the next generation sequencing technology (NGS), developed rapidly in the early twenty-first century and has been widely applied in various fields^8,9^. This technology can directly obtain sample DNA for library construction and sequencing analysis, and has advantages in analyzing microbial community structure.

Currently, more researches have focused on exploring the changes of microorganisms on the surface of tobacco leaves during fermentation, analyzing the community of microorganisms in the fermentation of tobacco leaves^10–13^. However, the effect of flue-curing and redrying processes on the microorganisms is ignored, which may lead to a longer fermentation period and slower quality improvement of tobacco leaves^6^. There are very few studies on the effects of flue-curing and redrying processes on the bacterial community on the surface of tobacco leaves. Ye et al. used the DGGE to study the change of bacterial populations on the leaf surface of tobacco. They found that the redrying process could reduce the bacterial population on the surface of tobacco leaves, which may affect the fermentation process^6^. Their further study showed that the bacterial communities also largely differed between raw and redried tobacco leaves. *Proteobacteria* was the most dominant phylum (56.15%) on raw tobacco leaves and *Firmicutes* (76.49%) was the most dominant phylum on redried tobacco leaves based on Illumina sequencing^7^. These studies mainly focused on the changes in bacterial diversities on the leaf surface before and after redrying and during the aging process. This study aims to systematically investigate the distribution and dynamics of bacteria communities on the surface of tobacco leaves during the processing stage from flue-curing to redrying. Different grades of tobacco leaves from different regions were used to study the differences in bacterial community structure before and after flue-curing and redrying processing stages, providing evidence for improving tobacco leaf quality artificially.

## Materials and methods

### Materials

Tobacco samples of four tobacco varieties which are typical varieties commonly used in cigarette formulations, including *Nicotiana tabacum* L. Yunyan 87, CB-1, Zhongyan Texiang 301, and Qinyan 96, were collected from tobacco-planting-fields, flue-curing houses, and redrying factories in seven domestic production areas, including Lufeng (Yunnan), Bozhou (Guizhou), Ninghua (Fujian), Huidong (Sichuan), Guiyang (Hunan), Mianchi (Henan) and Feixian (Shangdong) at four processing stages (before flue-curing, after flue-curing, before redrying, and after redrying). Three representative samples were collected for each group at different processing stages. A total of 36 samples were collected.

### DNA extraction, PCR amplification and high throughput sequencing

Total microbial genomic DNA of tobacco leaf samples was extracted using the CTAB method^14^. The purity and concentration of genomic DNA were assessed using agarose gel electrophoresis and the NanoDrop2000 (Thermo Fisher Scientific, Waltham, MA, United States). The DNA concentration of each sample was diluted to 1 ng/μL. For PCR amplification, the bacterial V4 region of the 16S rRNA gene was amplified by PCR (98℃ for 1 min, 30 cycles at 98℃ for 10 s, 50 °C for 30 s, and 72 °C for 30 s, and, finally, an extension at 72 °C for 5 min) using primers 515F (5′-GTGYCAGCMGCCGCGGTAA-3′) and 806R (5′-GGACTACHVGGGTWTCTAAT-3′)^15^. Each PCR reaction consisted of a 30 μL mixture containing of 15 μL of Phusion® High-Fidelity PCR Master Mix (New England Biolabs, United States), 0.2 μL of each primer (1 μM), and 10 ng of template DNA. The PCR products were detected with 2% agarose gel electrophoresis and purified with the Qiagen Gel Extraction Kit (Qiagen, Germany). Libraries were constructed as described in NEBNext® Ultra™ IIDNA Library Prep Kit protocol (New England Biolabs, United States). Finally, the libraries were loaded on an Illumina NovaSeq 6000 platform (Illumina, United States).

### Bioinformation analysis

Sequencing was performed using a paired-end configuration. The multiplexed amplicon samples were sequenced using Illumina MiSeq system using the MiSeq Reagent Kit V3, 600 Cycles (Illumina Inc., San Diego, CA, USA), following the default standard procedures. Each de duplication sequence generated after using DADA2^16^ method of QIIME (version 2) was called Amplicon Sequence Variables (ASVs)^17^. In QIIME (version 2) analysis, DADA2 software was used for quality control. Each ASV was annotated with species using the classify-sklearn algorithm of Quantitative Insights Into Microbial Ecology (QIIME, version 2)^18^. Mitochondria and chloroplast sequence contamination of host tissue in 16S ribosomal RNA gene (16S) analyses were removed according to sequence identity. All alpha indices of bacterial communities were calculated with QIIME (version 2). The beta diversity of UniFrac distance was calculated with QIIME (version 2) and displayed with R software. Linear discriminant analysis effect size (LEfSe) analysis was used to reveal the significant ranking of biomarkers between samples with a threshold of 3.0 in the logarithmic LDA score. Phylogenetic Investigation of Communities by Reconstruction of Unobserved Stats 2 (PICRUSt2) was used to predict metabolic function of bacterial communities^19^.

### Plant material

The collection of plant material complied with relevant institutional, national, and international guidelines and legislation. Liwei Hu was responsible for the formal identification of the plant material used in this study. Tobacco leaf materials were not deposited in the publicly available herbarium.

## Results

### High throughput sequencing analysis

As shown in Table 1, through bacterial 16S rDNA sequencing of 36 samples from different processing stages, a total of 4,490,040 valid sequences were obtained for further analysis, with an average of 124,723 sequences per sample. The average length of sequences was 375 bp. A total of 33 phyla, 79 classes, 195 orders, 344 families, 826 genera, and 7922 amplicon sequence variants (ASVs) were identified in the bacterial communities of tobacco leaves.

**Table 1:** Sequence data analysis and diversity index of samples in tobacco leaves of different processing stage.

Sample	Sequence	Average length (bp)	Shannon	Simpson	Chao	Coverage
Before flue-curing	1 219 235	376.33 ± 0.87	5.76 ± 1.31	0.92 ± 0.05	418.10 ± 229.84	0.99 ± 0.01
After flue-curing	1 373 965	375.33 ± 1.94	6.49 ± 0.95	0.95 ± 0.03	604.03 ± 158.00	0.98 ± 0.01
Before redrying	918 588	375.67 ± 1.32	6.17 ± 1.00	0.94 ± 0.04	481.65 ± 157.62	0.99 ± 0.01
After redrying	978 252	372.67 ± 3.91	7.15 ± 0.62	0.97 ± 0.02	793.38 ± 121.01	0.98 ± 0.00
Total	4 490 040	–	–	–	–	–

The data in the table are expressed as mean and standard deviation.

**Figure 1:**
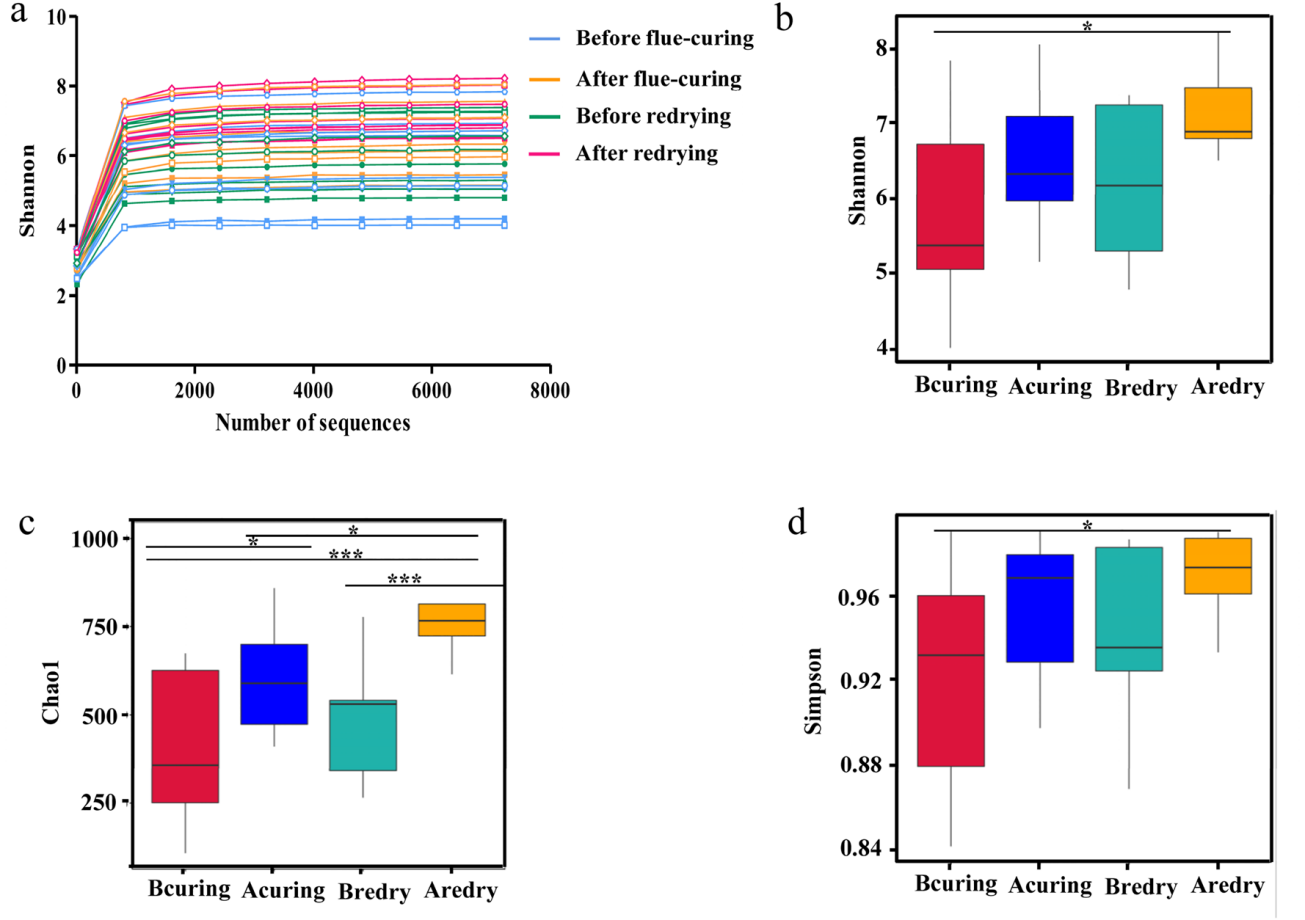
Dilution curves of sequenced tobacco leaf samples (**a**) and box plots of shannon index (**b**), chao1 index (**c**) and simpson index (**d**) between groups. “*” represents a statistical difference between groups (*p* < 0.05, Wilcoxon Signed Rank Test) and “***” represents a significant statistical difference between groups (*p* < 0.001, Wilcoxon Signed Rank Test). Bcuring, Before flue-curing; Acuring, After flue-curing; Bredry, Before redrying; Aredry, After redrying.

According to Shannon indexes of all samples, the dilution curves at different processing stages were obtained, as shown in Fig. 1. As the number of sample sequences increased, the dilution curves basically achieved saturation phases, indicating that the sequencing was found to reflect the bacterial diversity in tobacco leaf samples before and after flue-curing and redrying. Based on the analysis of richness and diversity of bacterial in all samples, the Shannon and Simpson values ranged from 5.76 to 7.15 and 0.92 to 0.97, respectively. The Chao values reflecting community richness ranged from 418.10 to 793.38. After flue-curing and redrying, the shannon index, chao1 and simpson index of the bacterial communities were increased (Fig. 1b–d). The sample coverage index at different processing stages was above 0.98, indicating that the sequencing was deep enough to represent all bacterial communities (Table 1).

### Bacterial community structure

**Figure 2:**
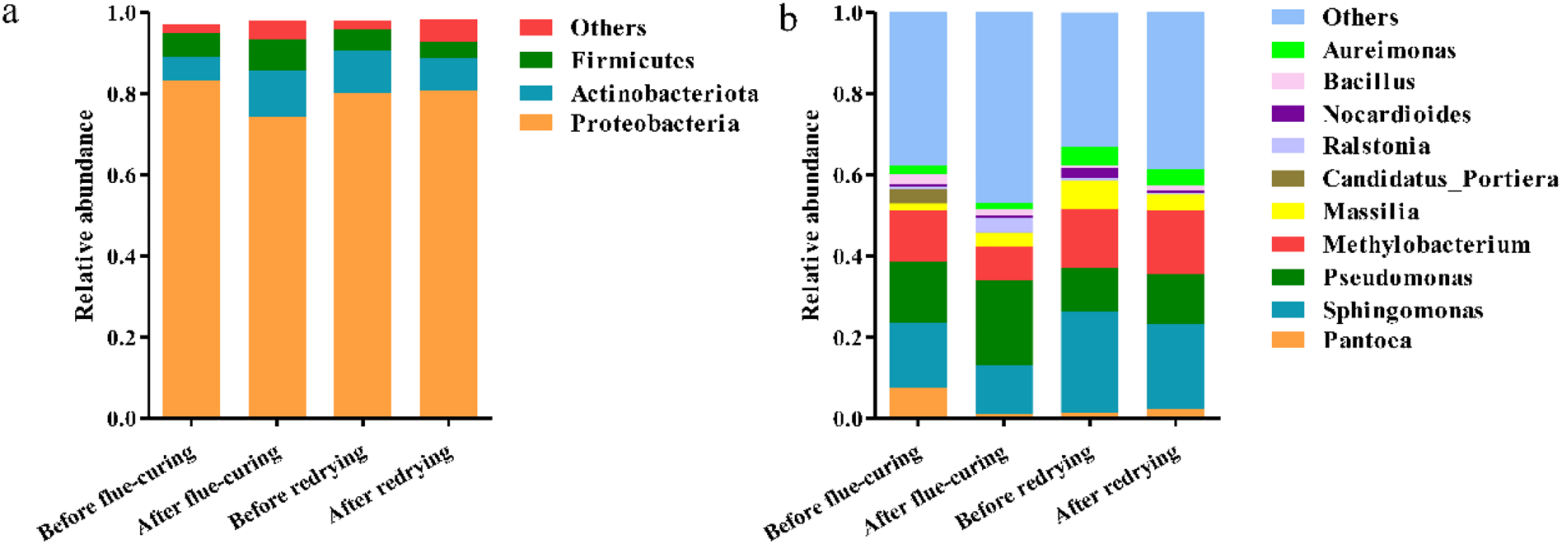
The relative abundance of bacteria at the level of phylum (**a**) and genus (**b**) in tobacco leaves of different processing stage.

The bacterial community structure of tobacco leaf samples at different processing stages were analyzed. The sequences were classified into the levels of phylum and genus in Fig. 2. A total of 33 phyla were identified in all tobacco leaf samples. *Proteobacteria*, *Actinobacteriota* and *Firmicutes* were the main phyla common to all samples. *Proteobacteria* was found to be the dominant phylum at four processing stages. The relative abundance of *Actinobacteriota* increased from 5.74% before flue-curing to 8.03% after redrying, and the relative abundance of *Firmicutes* decreased from 6.03% before flue-curing to 4.00% after redrying.

Bacterial community at the genus level was shown in Fig. 2b, a total of 826 genera had been identified in all tobacco leaf samples, of which *Sphingomonas*, *Pseudomonas*, and *Methylobacterium* were the prevalent genera common to all samples. The relative abundance of *Sphingomonas* was 15.81% before flue-curing, decreased to 11.81% after flue-curing, and decreased to 20.77% after redrying. The relative abundance of *Pseudomonas* increased from 15.23% before the flue-curing to 21.10% after the flue-curing, and decreased to 12.46% after the redrying. The relative abundance of *Methylobacterium* decreased from 12.68% before the flue-curing to 8.21% after the flue-curing, and increased to 15.64% after the redrying (Table S1). *Sphingomonas*, *Pseudomonas*, and *Mycobacterium* all belong to the *Proteobacteria*.

### Unique and shared ASVs analysis

**Figure 3:**
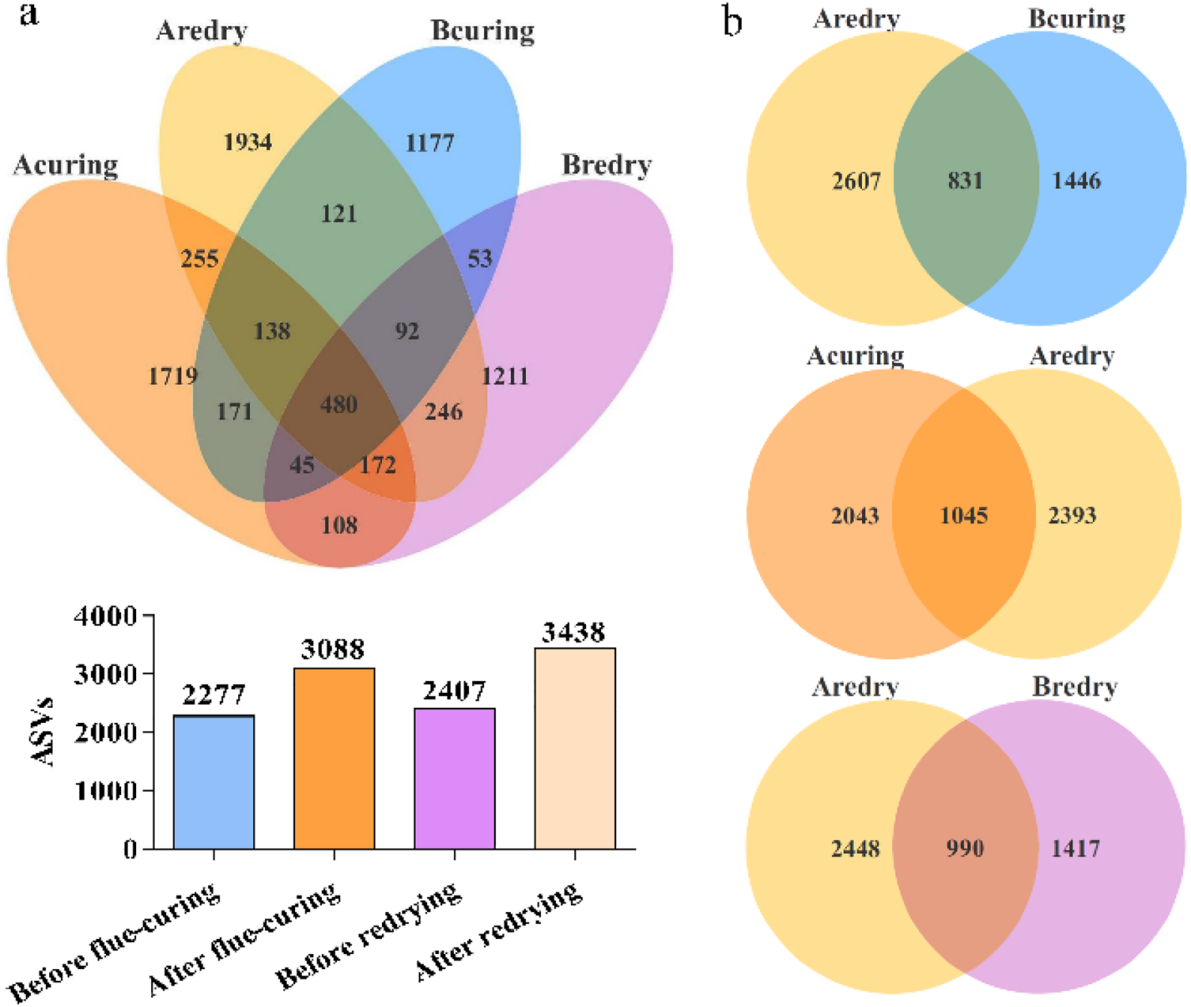
Venn diagram showing tobacco leaf ASVs distribution at different processing stages. Each circle in venn diagram represents a sample (group). Numbers in the non-overlapping region indicate unique ASVs for the single sample; numbers in the overlapping region indicate shared ASVs for multi-samples. The orange, yellow, blue and purple circles in the figure represent the stages of after flue-curing, after redrying, before flue-curing and before redrying, respectively. Bcuring, Before flue-curing; Acuring, After flue-curing; Bredry, Before redrying; Aredry, After redrying.

Next, the bacterial unique and shared ASVs were analyzed in tobacco leaf samples at different processing stages. As shown in Fig. 3, there were differences in bacterial species at four processing stages. A total of 480 shared ASVs accounted for 6.06% of all ASVs were found at four stages. Compared with before flue-curing, the number of ASVs increased after flue-curing, before redrying, and after redrying by 35.6%, 5.71%, and 50.99%, respectively. There were 1177, 1719, 1211, and 1934 unique ASVs in samples at 4 processing stages of before flue-curing, after flue-curing, before redrying and after redrying, respectively, accounting for 14.86%, 21.70%, 15.29%, and 24.41% of all ASVs, with the highest number of unique ASVs after redrying (Fig. 3a). The bacterial community on tobacco leaves after redrying affect the aging quality. The number of ASVs shared in samples between before flue-curing and after redrying, after flue-curing and after redrying, before redrying and after redrying was 831 (17.01%), 1045 (19.07%), and 990 (20.39%), respectively. The higher number of shared ASVs in samples after flue-curing and after redrying indicating that the increased bacterial community on the leaf surface during the flue-curing process had a significant impact on the bacterial richness in tobacco leaves after redrying (Figs. 1c, 3b).

### Differential species analysis

**Figure 4:**
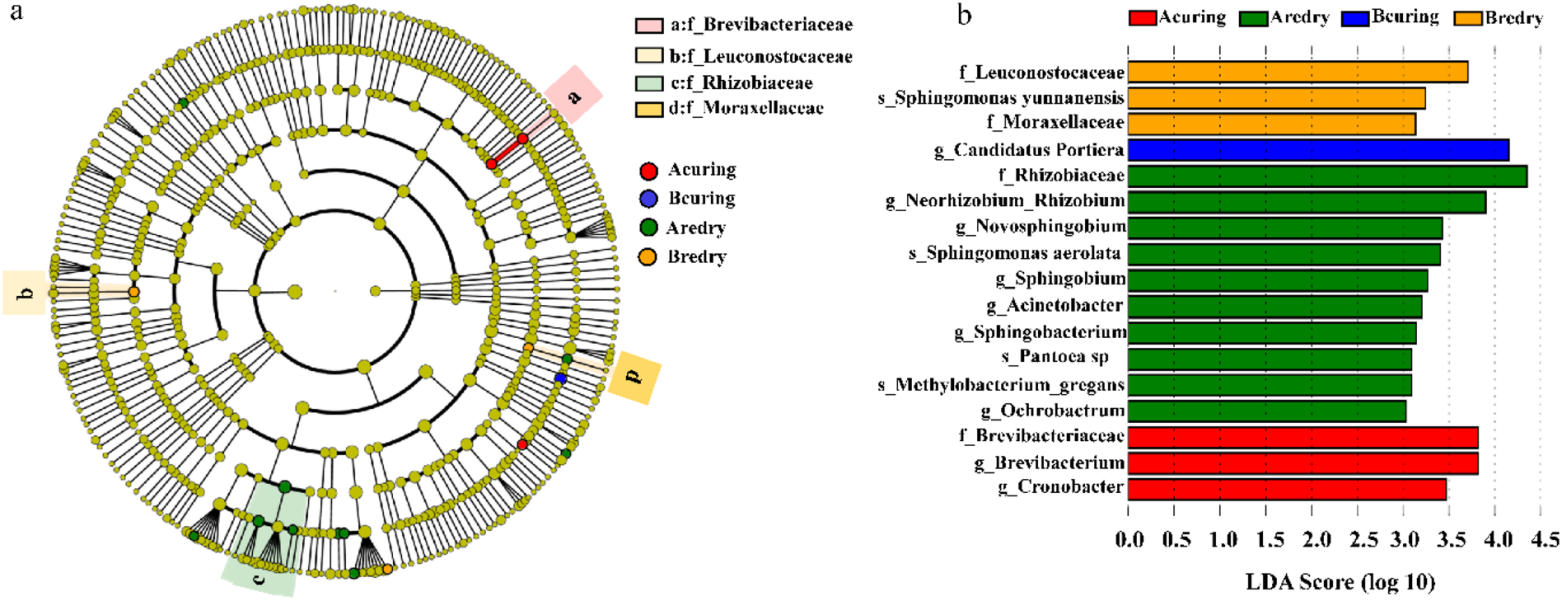
LEfSe analysis of bacterial communities among tobacco leaves at different processing stages. (**a**) In the cladogram, the circle radiating from inside to outside represents the classification level from phyla to genus (or species). The microbial groups which are not significantly different between groups were colored as yellow. (**b**) In the LDA score histogram, the lowercase letters represent difference indicator species, of which “f” represents family, “g” represents genus and “s” represents species. Bcuring, Before flue-curing; Acuring, After flue-curing; Bredry, Before redrying; Aredry, After redrying.

The analysis above showed that there were some differences in bacterial species in tobacco samples at different processing stages. Next, the species with significant differences in abundance at different processing stages were analyzed by LEfSe, and only those with LDA score values greater than 3 were shown (Fig. 4). The results suggested that 4 bacterial groups differed at the family level, namely *Brevibacteriaceae* after flue-curing, *Leuconostocaceae* and *Moraxellaceae* before redrying, and *Rhizobiaceae* after redrying. There were 9 different bacterial groups at the genus level, namely *Candidatus Portiera* before flue-curing, and *Rhizobiaceae* after redrying, *Portiera* before flue-curing, *Brevibacterium* and *Cronobacter* after flue-curing, and *Allorhizobium*-*Neorhizobium*-*Pararhizobium*-*Rhizobium*, *Novosphingobium*, *Sphingobium*, *Acinetobacter*, *Sphingobacterium* and *Ochrobactrum* after redrying.

### Bacterial community functional characteristics

**Figure 5:**
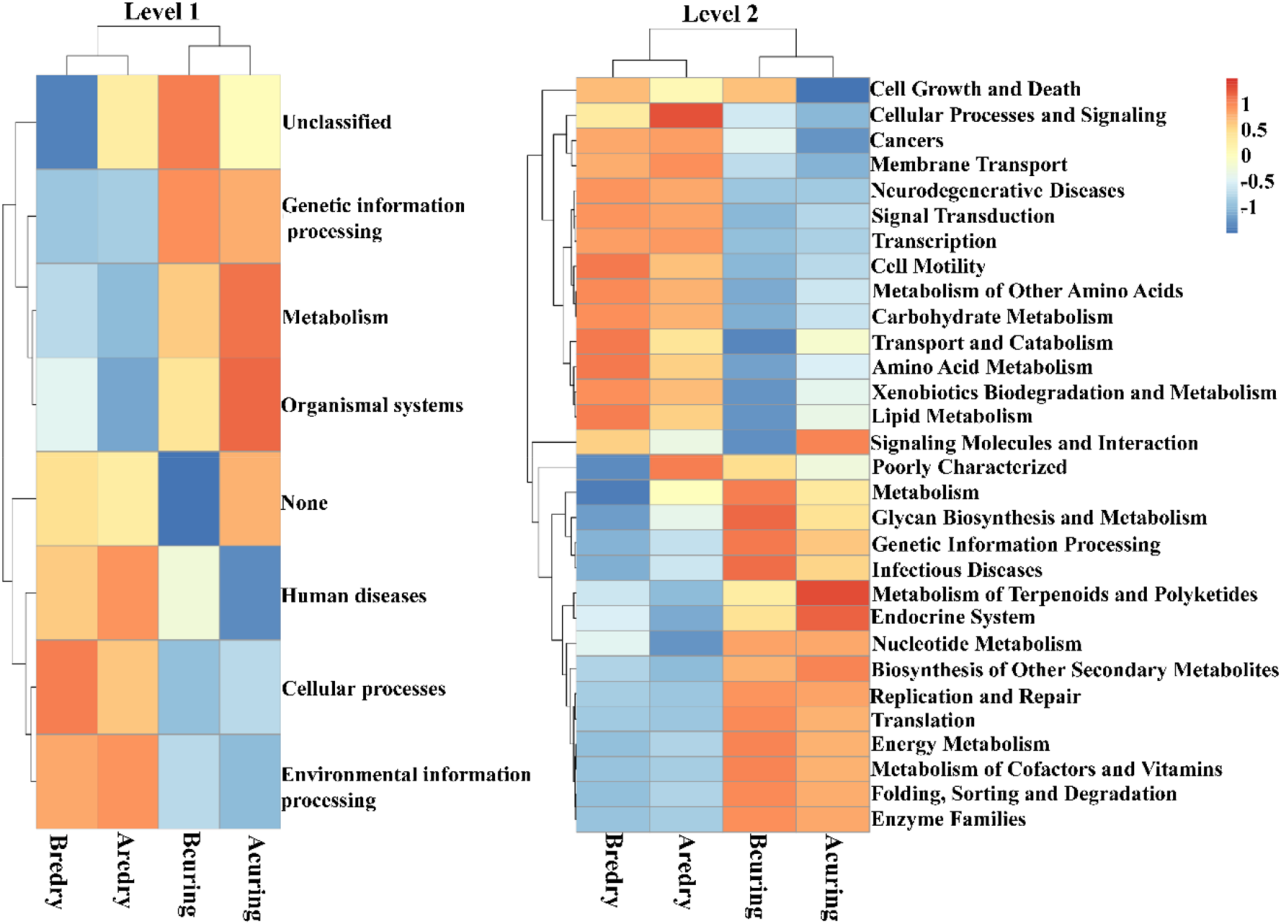
PICRUSt metabolic prediction of tobacco samples at different processing stages. Bcuring, Before flue-curing; Acuring, After flue-curing; Bredry, Before redrying; Aredry, After redrying.

PICRUSt was used for functional prediction of bacterial communities of tobacco samples. As shown in Fig. 5, with the processing of tobacco leaves, the metabolic functions of the tobacco bacterial community changed to adapt to the environmental changes. The metabolic pathways of the differential bacterial communities were analyzed at the primary and secondary functional levels, respectively. At the primary level, a total of 6 metabolic pathways were obtained, namely genetic information processing, metabolism, organismal systems, human diseases, cellular processes and environmental information processing. Metabolism, environmental information processing and genetic information processing were the major components, accounting for 47.34% ~ 49.21%, 15.30% ~ 17.23% and 14.40% ~ 15.47%, respectively. Among them, the highest relative abundance of metabolism pathway was after flue-curing (49.21%), the highest relative abundance of environmental information processing pathway was after redrying (17.23%), and the highest relative abundance of genetic information processing pathway was before flue-curing (15.47%). The secondary functional layer was also analyzed and consisted of 41 secondary functions such as membrane transport, amino acid metabolism, carbohydrate metabolism, energy metabolism, and replication and repair. After treatment of flue-curing, the relative abundance of pathways such as Xenobiotics biodegradation and metabolism and transport and catabolism increased, and metabolism of terpenoids and polyketides decreased significantly. After redrying, the relative abundance of pathways such as cellular processes and signaling, genetic information processing, glycan biosynthesis and metabolism increased, although there was no significant difference (Fig. 5, Supplementary Table 2).

## Discussion

The microbial community on the surface of tobacco leaves during the aging process is the main factor affecting the quality of tobacco leaves. Due to the fact that tobacco leaves must undergo flue-curing and redrying prior to aging, bacterial communities on tobacco leaves during four processing stages (before flue-curing, after flue-curing, before redrying, and after redrying) were systematically analyzed in this study. The diversity and structure of bacterial communities of tobacco leaves at different processing stages were analyzed by Illumina NovaSeq sequencing based on 16S rRNA genes. The analysis of microbial community structure showed that *Proteobacteria* was the most dominant phylum of samples from 4 processing stages (Fig. 2a). Most *Proteobacteria* microorganisms play an important role in the degradation and circulation of organic compounds. Similarly, Huang et al. and Su et al. reported that *Proteobacteria* was the dominant phylum of Zimbabwe and K326 flue-cured tobacco leaves^20,21^. Zhang et al.^8^ indicated that *Firmicutes*, *Proteobacteria*, *Actinobacteria* and *Bacteroidetes* were abundant in flue-cured tobacco leaves.

The dominant genera should be the main factor affecting the chemical composition of tobacco leaves. Further analysis of the bacterial communities at the genus level revealed that *Sphingomonas*、*Pseudomonas* and *Methylobacterium* were the main groups (Fig. 2b). *Pseudomonas* has the ability to degrade nicotine, and utilized nicotine as the sole carbon, nitrogen and energy, making it a dominant genus in tobacco leaves^22–24^. Therefore, *Pseudomonas* plays an important role in reducing tobacco smoking hazard. *Sphingomonas* can grow under high oxygen‐poor and harsh conditions^25^. *Sphingomonas* isolated from tobacco leaves has been reported to degrade polyphenols such as chlorogenic acid and widely used to improve smoke quality^26,27^. *Methylobacterium* is generally encountered as endophyte, which is used for biofilms formation and plant disease resistance^28,29^. Our results showed that the abundance of these dominant genera related to tobacco leaf quality varies among tobacco leaves at different processing stages, and different bacterial community composition may affect tobacco leaf quality.

The PICRUSt method was used to annotate the metabolic function of the identified microorganisms, and the results showed that there are differences in the metabolic functions of bacterial communities in tobacco leaves at different processing stages. High temperature and low water content during the flue-curing and redrying processes potentially affect bacterial communities and their metabolic pathways^7^. After treatment of flue-curing, the relative abundance of pathways such as Xenobiotics biodegradation and transport and catabolism increased, and metabolism of terpenoids and polyketides decreased significantly (Fig. 5, Supplementary Table 2). At this stage, fresh leaves undergo high-temperature dehydration to achieve rapid senescence. The significant increase of the Xenobiotics metabolism and catabolism after the treatment of flue-curing indicated that the microorganisms related to metabolism and catabolism accumulated on the surface of leaves after flue-curing can withstand the environment with low water content in the tobacco leaves, and may be responsible for compounds degradation in tobacco leaves during the subsequent fermentation process. Compared with before redrying, there was no significant difference in the relative abundance of metablic pathways after redrying. These results suggested that the flue-curing processing stage has a greater impact on the microorganisms on the leaf surface before aging, compared to the redrying processing stage. Similarly, the numbers of ASVs shared between before flue-curing and after redrying, after flue-curing and after redrying, before redrying and after redrying were 831 (17.01%), 1045 (19.07%), and 990 (20.39%), respectively, indicating that the flue-curing processing stage had a significant impact on bacterial communities in tobacco leaves after redrying (Fig. 3b). It is worth noting that after treatment of flue-curing, the relative abundance of metabolism of terpenoids and polyketides decreased significantly in Supplementary Table 2. Terpenoids are an important component of tobacco aroma compounds, which can improve the aroma quality of tobacco^30,31^. The decrease in the relative abundance of terpenoids metabolism after flue-curing indicated that the process conditions of flue-curing are an important factor affecting the aroma quality of tobacco.

In this study, we analyzed the effects of flue-curing and redrying on the structure of microbial communities on the surface of tobacco leaves and found that flue-curing may play an important role in influencing the quality of tobacco leaves. The chemical composition of tobacco leaves determines their quality. Therefore, we will focus on the changes in the chemical composition of tobacco leaves at different processing stages, establish a direct relationship between microbial community changes and tobacco quality, and obtain microorganisms with specific biological functions.

## Conclusions

In summary, bacterial communities in tobacco leaves were systematically analyzed through Illumina sequencing from flue-curing to redrying. *Proteobacteria* was the dominated phylum, and *Sphingomonas*, *Pseudomonas* and *Methylobacterium* were the main genera shared by all samples. Different bacterial diversities were observed in samples at four processing stages and contributed to differences in the metabolic related pathways. After flue-curing, the abundance of specific microorganisms related to metabolism such as Xenobiotics biodegradation and transport and catabolism significantly increased, and metabolism of terpenoids and polyketides decreased significantly. And these bacterial groups still maintained high relative abundance after redrying, suggesting that the flue-curing processing stage also had a significant impact on bacterial communities in tobacco leaves after redrying. This study provided insights into the importance of flue-curing in the tobacco leaf processing and indicated that the bacterial groups related to metabolism after flue-curing might have an effect on compounds degradation in tobacco leaves during the subsequent fermentation process.

### Supplementary Information

Supplementary Information 1.

Supplementary Information 2.

## Data Availability

The sequence reads generated and analyzed within this study are available on the National Center for Biotechnology Information Sequence Read Archive (BioProject PRJNA975704).
